# Identification of difficult laryngoscopy using an optimized hybrid architecture

**DOI:** 10.1186/s12874-023-02115-z

**Published:** 2024-01-04

**Authors:** XiaoXiao Liu, Colin Flanagan, Gang Li, Yiming Lei, Liaoyuan Zeng, Jingchao Fang, Xiangyang Guo, Sean McGrath, Yongzheng Han

**Affiliations:** 1 College of Mathematics and Information Science, Hebei University, Baoding, China; 2 Electronic and Computer Engineering, University of Limerick, Limerick, Ireland; 3 Department of General Surgery (GL), Peking University Third Hospital, Beijing, China; 4 Ministry of Education Engineering Research Centre on Mobile Digital Hospital Systems, School of Electronics, Peking University, Beijing, China; 5 School of Communications, University of Electronic Science and Technology of China, Chengdu, China; 6 Department of Radiology (JCF), Peking University Third Hospital, Beijing, China; 7 Department of Anaesthesiology, Peking University Third Hospital, Beijing, China

**Keywords:** Difficult laryngoscopy, Hybrid architecture, Atlantooccipital gap, Cervical spondylosis, Radiological variables

## Abstract

**Background:**

Identification of difficult laryngoscopy is a frequent demand in cervical spondylosis clinical surgery. This work aims to develop a hybrid architecture for identifying difficult laryngoscopy based on new indexes.

**Methods:**

Initially, two new indexes for identifying difficult laryngoscopy are proposed, and their efficacy for predicting difficult laryngoscopy is compared to that of two conventional indexes. Second, a hybrid adaptive architecture with convolutional layers, spatial extraction, and a vision transformer is proposed for predicting difficult laryngoscopy. The proposed adaptive hybrid architecture is then optimized by determining the optimal location for extracting spatial information.

**Results:**

The test accuracy of four indexes using simple model is 0.8320. The test accuracy of optimized hybrid architecture using four indexes is 0.8482.

**Conclusion:**

The newly proposed two indexes, the angle between the lower margins of the second and sixth cervical spines and the vertical direction, are validated to be effective for recognizing difficult laryngoscopy. In addition, the optimized hybrid architecture employing four indexes demonstrates improved efficacy in detecting difficult laryngoscopy.

**Trial registration:**

Ethics permission for this research was obtained from the Medical Scientific Research Ethics Committee of Peking University Third Hospital (IRB00006761-2015021) on 30 March 2015. A well-informed agreement has been received from all participants. Patients were enrolled in this research at the Chinese Clinical Trial Registry (http://www.chictr.org.cn, identifier: ChiCTR-ROC-16008598) on 6 June 2016.

## Introduction

Difficulty in airway management is one of the leading causes of anaesthesia-related cardiac arrest, death, and brain damage in patients undergoing surgery [1]. Unanticipated difficult airway has always been an issue even for experienced anaesthesiologists [2]. Adequate evaluation of airway structures prior to surgery is essential for safe and effective tracheal intubation, which is a prerequisite for safe anaesthesia, minimising distress, and reducing the need for additional sedation for reintubation [3]. Clinically, many factors are associated with difficult laryngoscopy, including head-neck trauma [4], airway abnormalities [5], gastroesophageal reflux disease [6], difficulty in opening mouth [7], impaired cervical mobility [8]. To achieve optimal laryngoscopic views, the oral, pharyngeral and laryngeal axes need to guarantee closest match, creating feasibility for anaesthesiologists to expose the glottis in patients with cervical spondylosis [7]. However, gold standards and guidelines have not been laid out for difficult laryngoscopy detection. The Mallampati classification predicts intubation ease based on oral cavity visibility, however, despite suggested modifications, adding neck mobility and mandibular space, it has limitations due to subjectivity and an inadequate assessment of airway problems.

Recent technological advancements in artificial intelligence (AI) algorithms, computer hardware, and large medical imaging datasets have enabled computer scientists and healthcare researchers to collaborate closely to improve airway management [9] and laryngoscopy prediction [10–12]. As a potent subfield of AI, deep learning has the potential to analyse large medical databases in parallel [13] via its multiple computational nodes and to identify potential intubation hazards via its hidden layers [14]. As one of the popular deep learning applications, vision transformers employing self-attention structures are able to extract global information, recognise patterns, and capture long-distance relationships from images, enabling efficient imaging detection, such as difficult laryngoscopy identification.

This study aims to identify difficult laryngoscopy using a novel attention-based AI model on a large preoperative X-ray dataset, as well as to investigate the relationship between image-measured indicators and difficult laryngoscopy. Two new indicators will be introduced to reflect the range of motion of the upper and lower cervical spine in relation to difficult laryngoscopy, and their efficacy will be evaluated by comparing them to two published indicators. Combining convolutional neural networks, spatial extraction, and vision transformer structures, this study will introduce a novel AI architecture for the identification of difficult laryngoscopy. In order to enhance the performance of the hybrid AI model, an attention-based spatial extraction structure will be implemented and its optimal placement will be determined. For the first time, the performance of difficult laryngoscopy prediction using a single index and using multiple indexes will be validated uniformly. The ranking of the four indicators will also be provided.

## Material and methods

### Datasets and samples

This prospective cohort study recruited patients undergoing elective cervical spine surgery under general anaesthesia during the period June 2016 to December 2021. The following criteria were included: (1) Age range of 20 to 70 years, (2) Psychiatric health, and (3) Intact radiological and medical records. Rule-out conditions were as below. (1) Airway neoplasm or foreign objects (tumours of the larynx, pharynx, tongue, floor of the mouth, or cysts involving the mandible or medial neck), (2) Serious cervical vertebral trauma, (3) Cervical instability, (4) Unstable physical condition (ASA IV or V), and (5) Anticipated difficulty with facemask ventilations (previous surgical intubation difficulties, surgery, neck radiotherapy). Ethics permission for this research was obtained from the Medical Scientific Research Ethics Committee of Peking University Third Hospital (IRB00006761-2015021) on 30 March 2015. A well-informed agreement has been received from all participants. Patients were enrolled in this research at the Chinese Clinical Trial Registry (http://www.chictr.org.cn; identifier: ChiCTR-ROC-16008598) on 6 June 2016.

Routine preoperative monitoring of non-invasive blood pressure, heart rate, pulse oximetry, and electrocardiography was performed. Sufentani (0.3 $\mu$g/kg) and propofol (2 mg/kg) were administered to induce anaesthesia. In unconscious patients, neuromuscular blockade was induced by rocuronium (0.6 mg/kg). The difficulty of laryngoscopy was ascertained by the single advanced anaesthesiologist using the Cormack-Lehane scales with the Macintosh laryngoscope for all participants in the olfactory position (Table 1) [15]. The anaesthesiologist was not engaged in perioperative radiographic evaluations. Those with grade III or IV views were allocated to the difficult laryngoscopy category, and those who had grade I or II views were allocated to the simple laryngoscopy category. Patients who were unsuccessful with the Macintosh laryngoscope were addressed in accordance with the Difficult Airway Society 2015 guidelines [16]. No patients were involved in the radiological data measurements nor were they involved in developing plans for the design and accomplishment of the present study. None of the patients was asked to advise on the interpretation. The radiology staff was blinded to the examination. The results will be disseminated to investigators and patients through this publication.

**Table 1:** The Cormack Lehane (C-L) scale

Classification	Description
Class I	Vocal cords were completely visible
Class II	The arytenoids were visible
Class III	Only the epiglottis was visible
Class IV	The epiglottis was not visible

A previous study showed that the incidence of difficult laryngoscopy was 24% [17]. In our preliminary study, the incidence of difficult laryngoscopy was 18.6%. It was estimated that the sample sizes of 610 would achieve to detect a difference in indicators between the difficult and easy laryngoscopy groups ($\alpha$ = 0.05 and $\beta$ = 0.1), and in consideration of 10% dropout rate, 671 patients were enrolled in this study.

### Data pre-processing

**Fig. 1:**
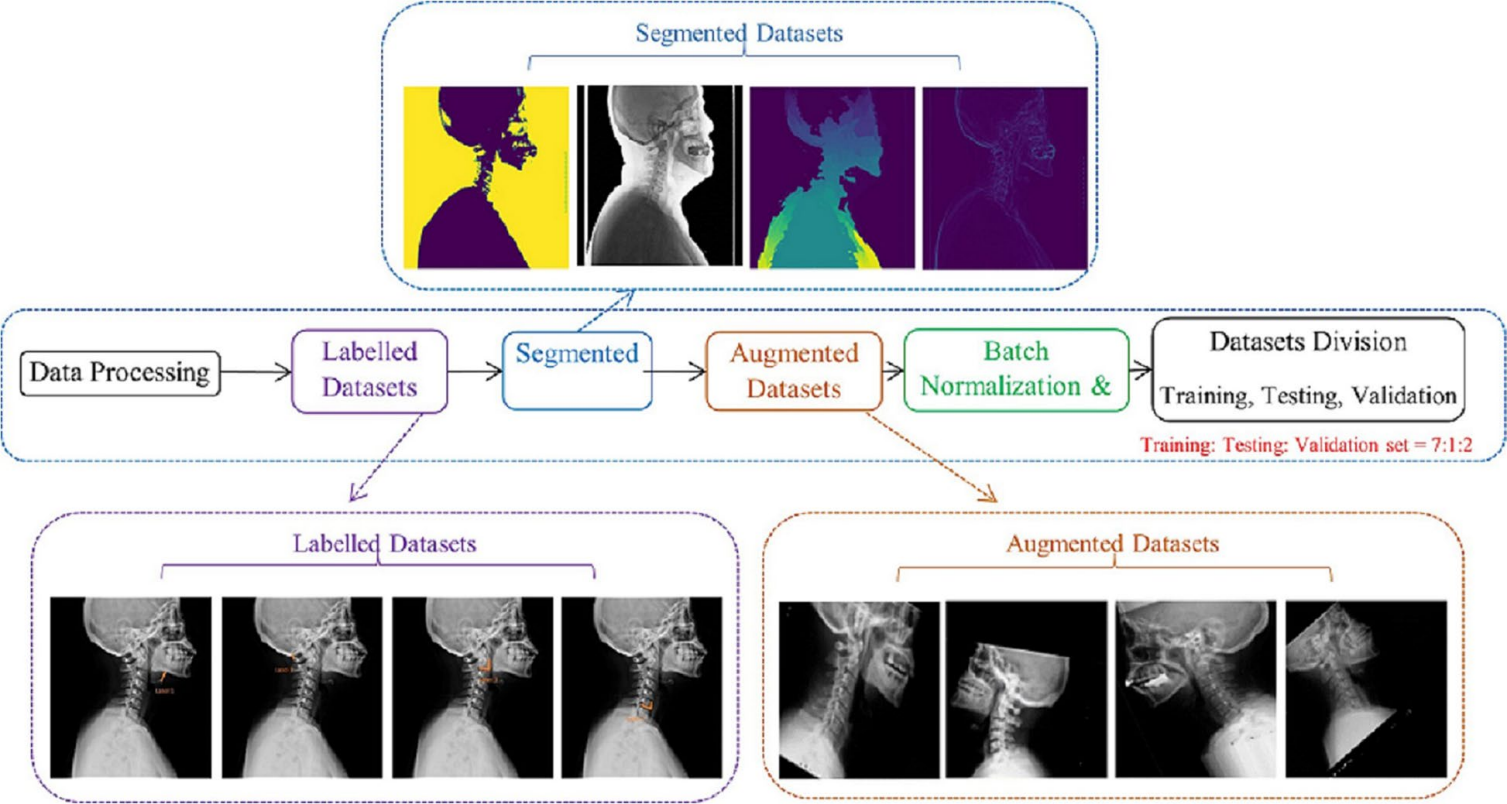
Dataset processing, including data labelling, data segmentation and data augmentation

**Fig. 2:**
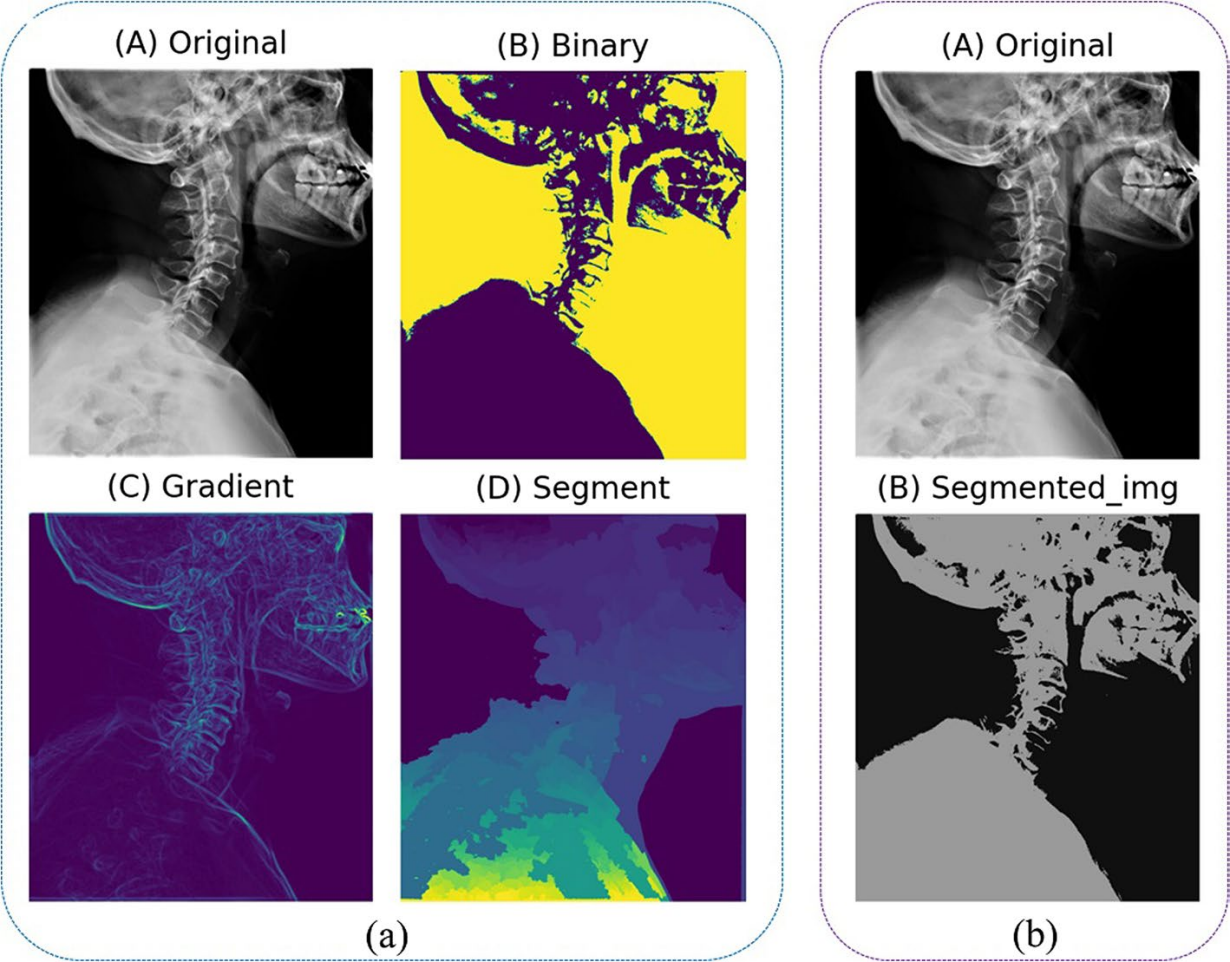
Hybrid segmentation

Patients’ clinical and radiological information was obtained from their medical records and the image archiving and communication system (PACS). Pre-processing is a crucial step in medical image classification, especially in the processing of large datasets. AI techniques for medical images typically rely on supervised learning, utilizing datasets containing data points (e.g., images) and labels (e.g., object classes) [13]. Pre-processing including data segmentation, labeling, data enhancement [18], and data balancing was mainly performed on histograms and labelled images in this study (Fig. 1). Feature extraction is vital for image classification. Medical image segmentation is one of the most promising methods in medical image analysis, which identifies pixels of organs or lesions from backgrounds such as X-ray images, providing critical morphologic and spatial information of these images [19]. In this study, hybrid segmentation methods were utilized to extract characteristics and enhance the imaging detection performance, including grayscale conversion, binary transformation, skeleton extraction, central axis transformation, gradient extraction, and K-means method (Fig. 2). In (a), images were segmented using the watershed algorithm.

(A) The original image.
(B) The original image after binarization. The threshold ranged from 90 to 255, and the pixel value 255 became 1 after binarization.
(C) The gradient of the image obtained after noise filtering, using gradients below 10 as the starting gradient points.
(D) The gradient and marker information are used to generate a gradient-based watershed map.

In (b), images were segmented using the K-means clustering algorithm.

(A) The original image.
(B) This image was obtained using K-means clustering. CNNs have achieved leading-edge capability in numerous biomedical image categorization assignments [20] including diverse modalities [21]. They require a richly tagged database, where the category of each of the pixels or voxels is known, to direct the processing of the database. Yet, collecting intensively tagged biomedical pictures is challenging, as tagged medical databases demand field-specific information and the pixel level annotations are potentially time consuming [22].

Data enhancement is an efficient method for increasing the number and diversity of datasets via stochastic moulding [23]; In the imaging field, commonly available augmentation techniques include image resizing, rescaling, and vertical rotation. In this research, rotational variation, breadth variation, altitude variation, stochastic clipping, scaling variation, and horizontal flipping were implemented to execute data augmentation. After data augmentation, each image was resized to 64 x 64 pixels for the CNN models and 180 x 180 pixels for the transfer learning models.

Data segmentation is a useful technique for removing superfluous features from difficult laryngoscopy images; however, poor contrast and imprecise brightness distributions in laryngoscopy imaging may result in unreliable segmentation [24]. Histogram equilibrium plays a crucial role in image quality enhancement [25], and it was utilised to mitigate such issues in this study [26]. Category weights are utilized during model training to evaluate the loss function. This manipulation enables the target model to ’give more consideration’ to samples from representative categories. The weights are based on the proportion of difficult and simple laryngoscopy.

### Proposed indexes

**Fig. 3:**
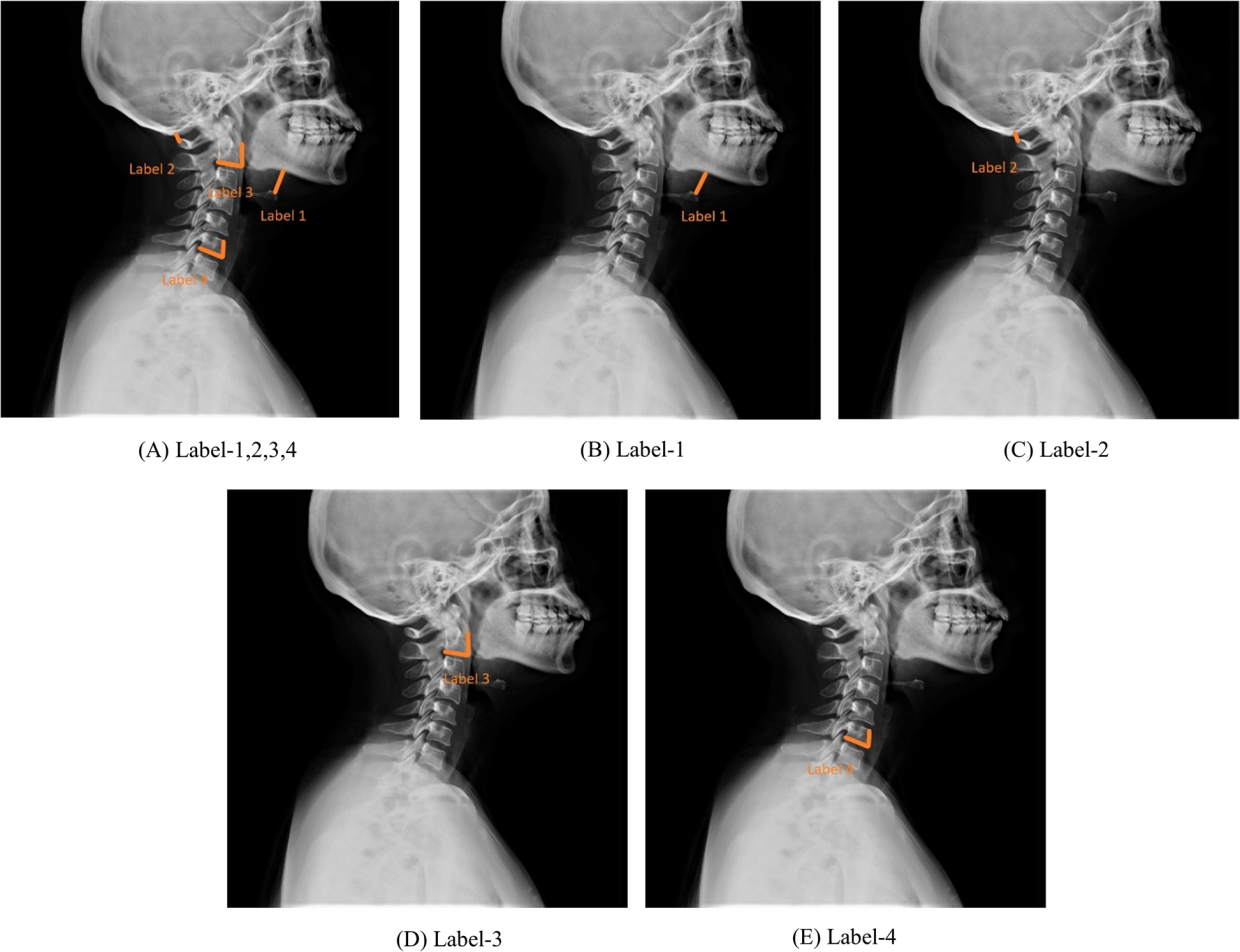
Labelled laryngoscope imaging

This study presents two new indicators that reflect the range of motion of the upper and lower cervical spine in relation to difficult laryngoscopy and compares them to two previously published indicators to determine their efficacy. Figure 3 depicts the two new indicators, Label-3 and Label-4, in addition to the two previously published indicators, Label-1 and Label-2. The first new indicator, Label-3, is the angle between the lower margin of the second cervical spine and the vertical direction. This measurement is of utmost importance because it reflects the degree of anterior laryngeal displacement, which is known to be associated with difficult laryngoscopy [27]. A smaller angle in this measurement indicates a higher likelihood of difficult laryngoscopy. The angle between the lower margin of the sixth cervical spine and the vertical direction is the another new indicator, Label-4. This measurement is crucial because it reflects the degree of cervical flexion, which can also contribute to difficult laryngoscopy. The likelihood of encountering difficult laryngoscopy increases as the angle of this measurement increases. Label-1: Vertical distance from the highest point of the hyoid bone to the mandibular body [28]. Label-2: Atlanto-occipital gap [29]. Label-3: The angle between the lower margin of the second cervical spine and the vertical direction, indicating the range of motion of the upper cervical spine [27]. Label-4: The angle between the lower margin of the sixth cervical spine and the vertical direction, indicating the range of motion of the lower cervical spine.

Each of the four indications pertains to cervical spine information; therefore, they are all intrinsically linked to the identification of a difficult laryngoscopy. No comparison of the four indicators was performed in previous studies. The work evaluated and contrasted the efficacy of four indicators and ranked them in order of efficacy. The neutral position was used for all cervical spinal x-rays.

### Classical architecture

Various advances in artificial intelligence (AI) are rapidly sweeping the medical imaging field. They have the ability to correctly interpret external data, draw experience and lessons from it, and adapt flexibly to achieve particular objectives [30]. The key deep learning technique leveraged in these tasks is the convolutional neural network (CNN), a type of deep learning algorithm that hardcodes translational invariance, which is a key feature of image data. CNNs have achieved extraordinary success in medical video classification and detection [31], medical image regression [32], medical image classification [33], medical image segmentation, or image registration tasks. This work applied a six-layer CNN model to identify difficult laryngoscopy. The implementation of maxpooling structures and sigmoid functions came after each CNN layer. At the end of the model, a dense layer was applied.

Many deep learning and data mining algorithms assume that the training data and the future real data must have the same characteristics and distribution. However, this assumption may not hold true in many real-world applications. Transfer learning models, in contrast, use different domains, tasks, and distributions for training and testing [34]. Pre-training on widely accepted large datasets, such as ImageNet or COCO, can improve the ability of artificial intelligence models to generalize to new medical imaging datasets. This work applied three different transfer learning model: DenseNet-121, ResNet-50, and VGG-16.

### Proposed hybrid structure

**Fig. 4:**
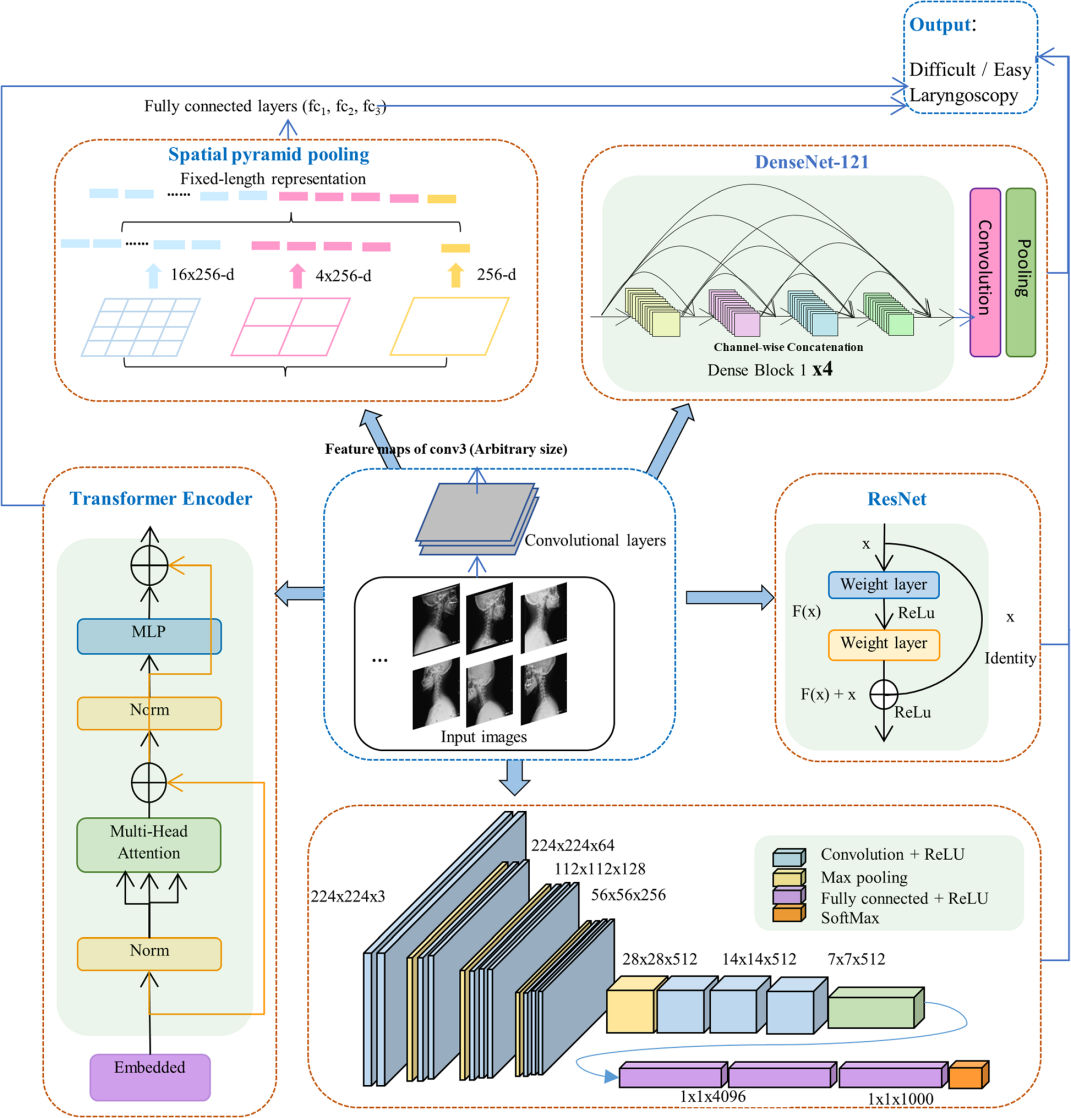
Adaptive multi model structure

This work proposes a hybrid artificial intelligence (AI) architecture for the analysis of laryngoscopy images that integrates Convolutional Neural Networks (CNN), spatial extraction, and Vision Transformers (ViT) with attention mechanism (MSCNN) (Fig. 4). The proposed architecture is made up of a CNN for low-level feature extraction, such as lines and blocks, a spatial extraction structure (STN) for capturing multi-scale information, and a ViT for high-level representation learning, such as cervical spine curvature. Specifically, vision transformers are designed to capture long-range dependencies and global context by calculating loss using positional and patch features from the transformer encoder layers, which has the potential to enhance the efficacy of difficult laryngoscopy prediction; the spatial extraction component can divide the input image into multiple regions and independently process each region, allowing the model to concentrate on relevant areas and increase its localization accuracy.

CNNs are capable of extraction and classification of features. However, they may not be able to capture long-range dependencies and contextual information in the images, which can limit their performance on more complex tasks, such as identification of difficult laryngoscopy imaging. The hybrid model proposed has the potential to enhance performance by leveraging the benefits of its AI structures, specifically by incorporating contextual information from the entire image and learning discriminative features from each structure. Four additional structures, CNN, DenseNet-121, ResNet-50, and VGG-16, were utilized to validate the MSCNN.

**Fig. 5:**
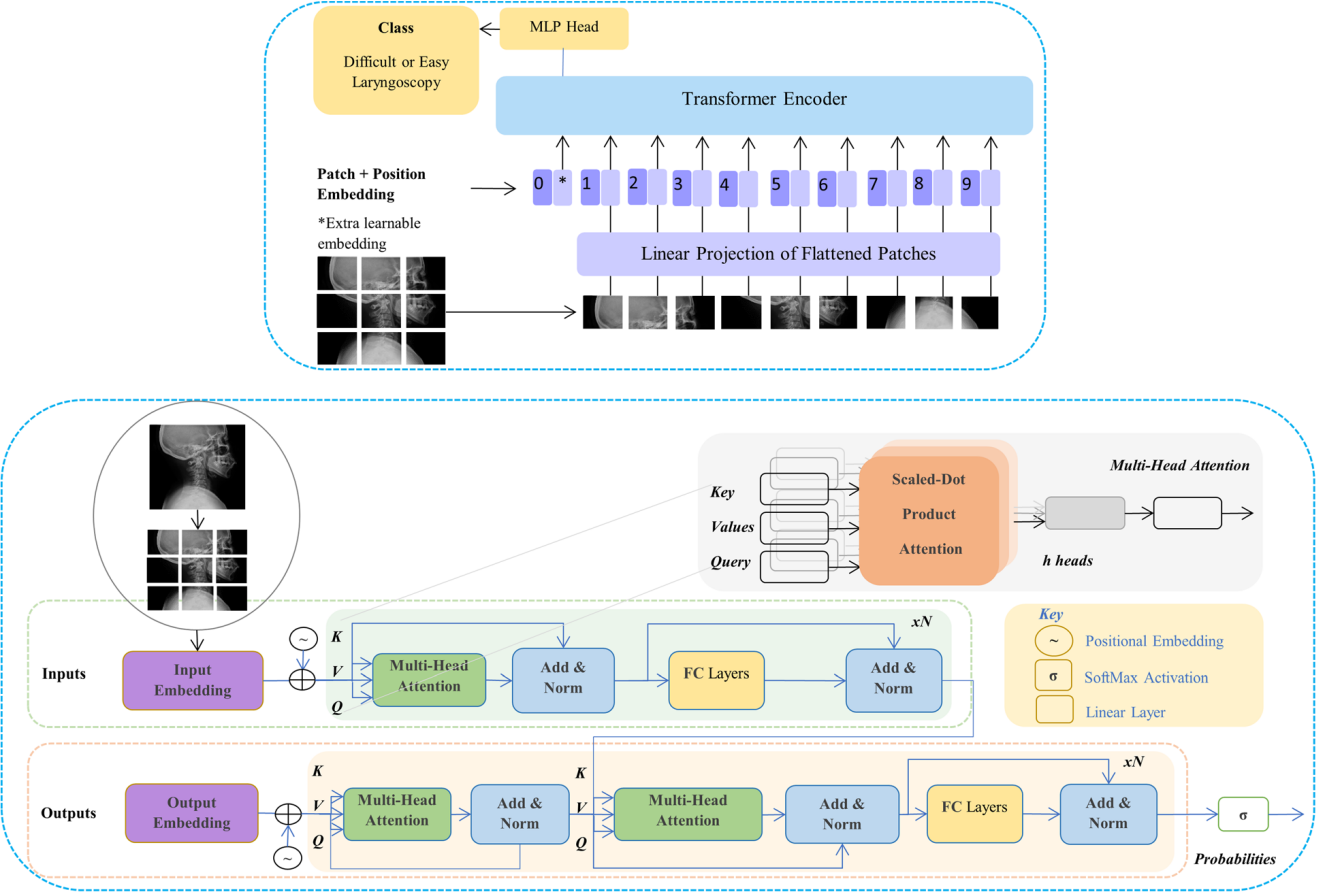
Visual multi head self attention transformer

The application of large CNN with many layers on a dataset will lead to over-fitting, that is, the models perform well on the training set, but with poor generalization ability. Therefore, it cannot predict on the unknown samples. This causes the CNN model to identify difficult laryngoscopy database of patients in the dataset used, but it cannot be generalized to identify whether other patient images are difficult. In this study, the method of learning employed by the model is adaptive, and its hypeparameters degrade automatically. If the loss value stays the same within ten steps, the early stop will be performed, and the learning rate will decrease. The resolution ratio of the square images of the training and validation sets was reduced to 64 x 64 pixels. The learning rate hyperparameter automatically decreases. Specifically, If the loss function does not decrease over three runs, the model will adapt and reduce the learning rate to 0.6 times, contributing to slower run times and more efficient models, and the model with the lowest loss will be selected. Vision Transformer has evolved into one of the most contemporary and predominant architectures in medical imaging. Transformer is a deep neural network based on a self-attention mechanism that facilitates substantially large receptive fields. It can capture global context with respect to CNN with local receptive fields [35]. Multi-head attention transformer, ViTBase16, was applied to capture global context of input images. Visual transfer models were utilized to improve the accuracy of difficult laryngoscopy detection. It first segments the imported picture to patches and casts the flattened ones into a feature space, which is processed by its encoder to generate the resulting classified outputs (Fig. 5).

### Optimization of proposed hybrid structure

**Fig. 6:**
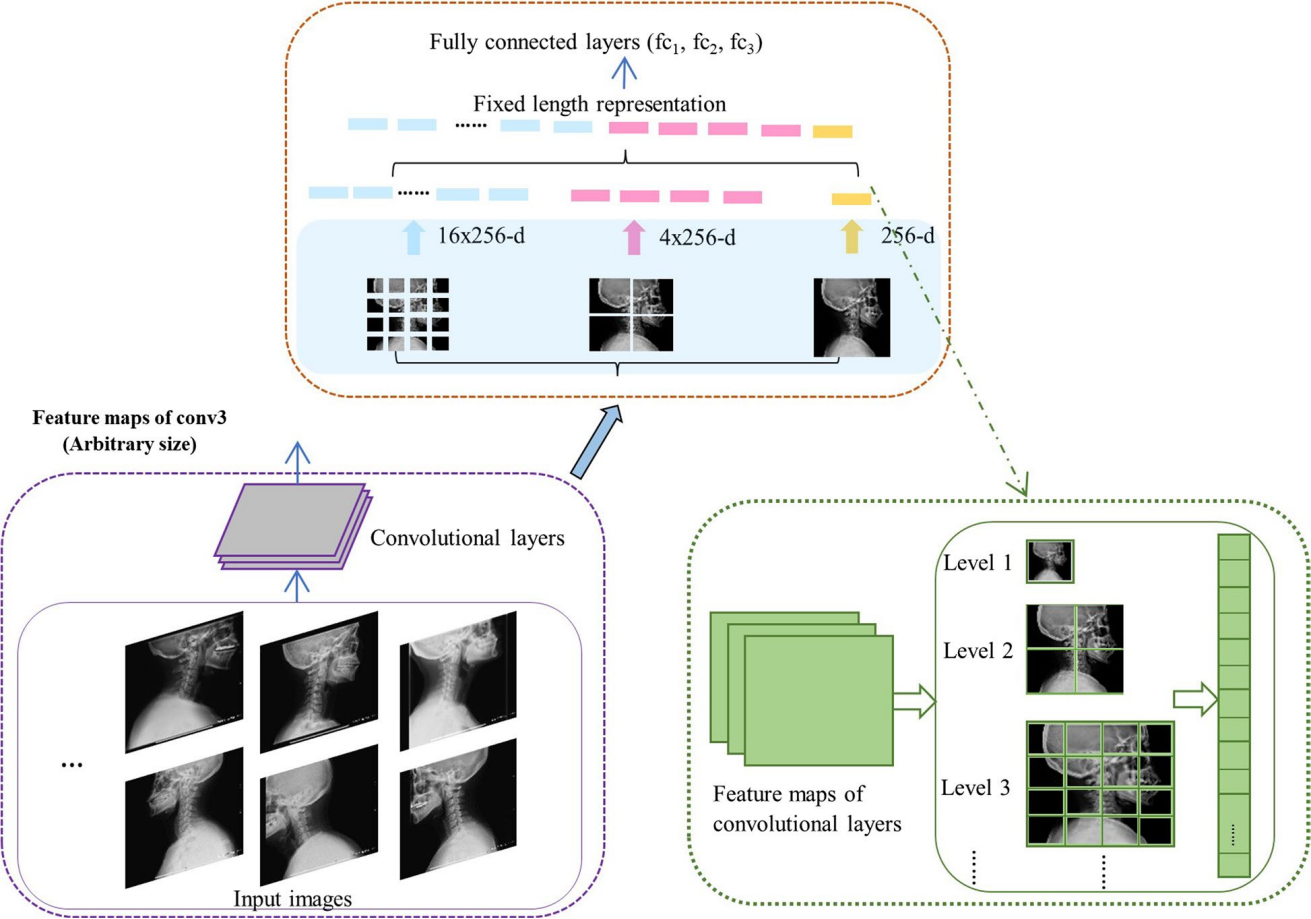
A network structure with a Fine-grained Spatial Interaction

AI is emerging as a formidable implement in the analysis of biomedical imaging, with deep learning employed to categorize airway prediction imaging for advanced detection of difficult laryngoscopy. Standard convolutional actions in deep learning do not expressly consider space-related interaction; therefore, space interactivity is considered as it has the potential to improve the efficiency of difficult laryngoscopy categorization by effectively preserving the spatial information of various scales prior to processing, resulting in robust information extraction. In this research, several granular spatial interaction structures were employed to eliminate the constant size limitation of this model. The spatial extraction structure was added on top of the final convolution layer, with the combination of three max-pooling structures positioned after the second, fourth, and sixth convolutional layers. The spatial extraction structure aggregates characteristics and produces a constant-length outcome, which is then sent to a fully concatenated layer (or another classifier). In other words, in order to avoid clipping or twisting at the beginning, certain “aggregation” is carried out at a higher stage of the model hierarchy (between convolution and fully connected hierarchies) [36] (Fig. 6) shows the structure of a Fine-grained Spatial Interaction. Here, 256 is the filter number of the last convolutional layer. In this study, three visual transfer learning methods (ResNet-50, DenseNet-121, and VGG-16) were used for feature extraction from the laryngoscopy images. All the transfer learning models were pretrained on the ImageNet dataset. The hidden deep layers used in the deep transfer learning map input data to indexes to analyse hidden patterns in complicated data.

Label-1,2,3,4 was used by all of the AI models in this study to validate their performance. Additionally, the following five different label image data were employed on DenseNet-121, CNN+SPP, and Hybrid Model, respectively, in order to completely verify the importance of various variables in discriminating difficult laryngoscopy: Label-1,2,3,4, Label-1, Label-2, Label-3, and Label-4.

### Statistical analysis

This research presents a comprehensive approach to binary classification using CNN, ResNet-50, VGG-16, DenseNet-121, CNN+SPP, and a hybrid model that incorporates both CNN, SPP and Vision Transformer (ViT). It compared various aspects, including deep learning techniques, evaluation metrics, Python implementation, and descriptive components such as loss functions, activation functions, optimizers, and model architecture.

This study leverages Python, a versatile programming language, and popular deep learning library, TensorFlow. It provides the necessary tools for model construction, training, and evaluation. For binary classification, this research employed binary cross-entropy. ReLU (Rectified Linear Unit) and sigmoid are utilized in this study, they are crucial for introducing non-linearity into the model. They enable the network to learn complex patterns and make predictions. Adam and SGD (Stochastic Gradient Descent) are used to update model parameters during training. The selection of an optimizer influences the convergence speed and final model performance.

Model performance is assessed using various evaluation metrics, including test accuracy and the average precision score. Test accuracy is a commonly used evaluation metric for classification models. It measures the proportion of correctly classified instances in the test dataset. In binary classification, it calculates the ratio of true positives (correctly predicted positive instances) and true negatives (correctly predicted negative instances) to the total number of instances in the test set. The average precision score was calculated in this research. It quantifies the area under the precision-recall curve (PR curve), which plots precision against recall for different classification thresholds. It provides a single value that summarizes the model’s ability to make precise positive predictions while considering all possible classification thresholds. The higher the average precision score, the better the model’s performance. To compute the average precision score, you can use libraries like Scikit-Learn in Python.

This research implements the binary classification model using Python code, leveraging deep learning libraries for model construction and training. Visualization libraries like Matplotlib and Seaborn are employed for result visualization.

## Results

### Trial setup

A total of 671 patients preoperative cervical spine X-ray images are collected and used in this study, including 548 easy and 123 difficult laryngoscopy patients. The processor uses GPUs. The optimal model is preserved by keeping the model checkpoint with the least loss value. The dataset is shuffled and divided into training, test, and validation sets in a ratio of 7:1:2. The resolution of each image of the training, test and validation sets is reduced to 224 x 224 pixels. The dimension of each image is 700 x 700 with a bit depth of 8.

### Accuracy of classical structures using proposed indexes and classical indexes

**Fig. 7:**
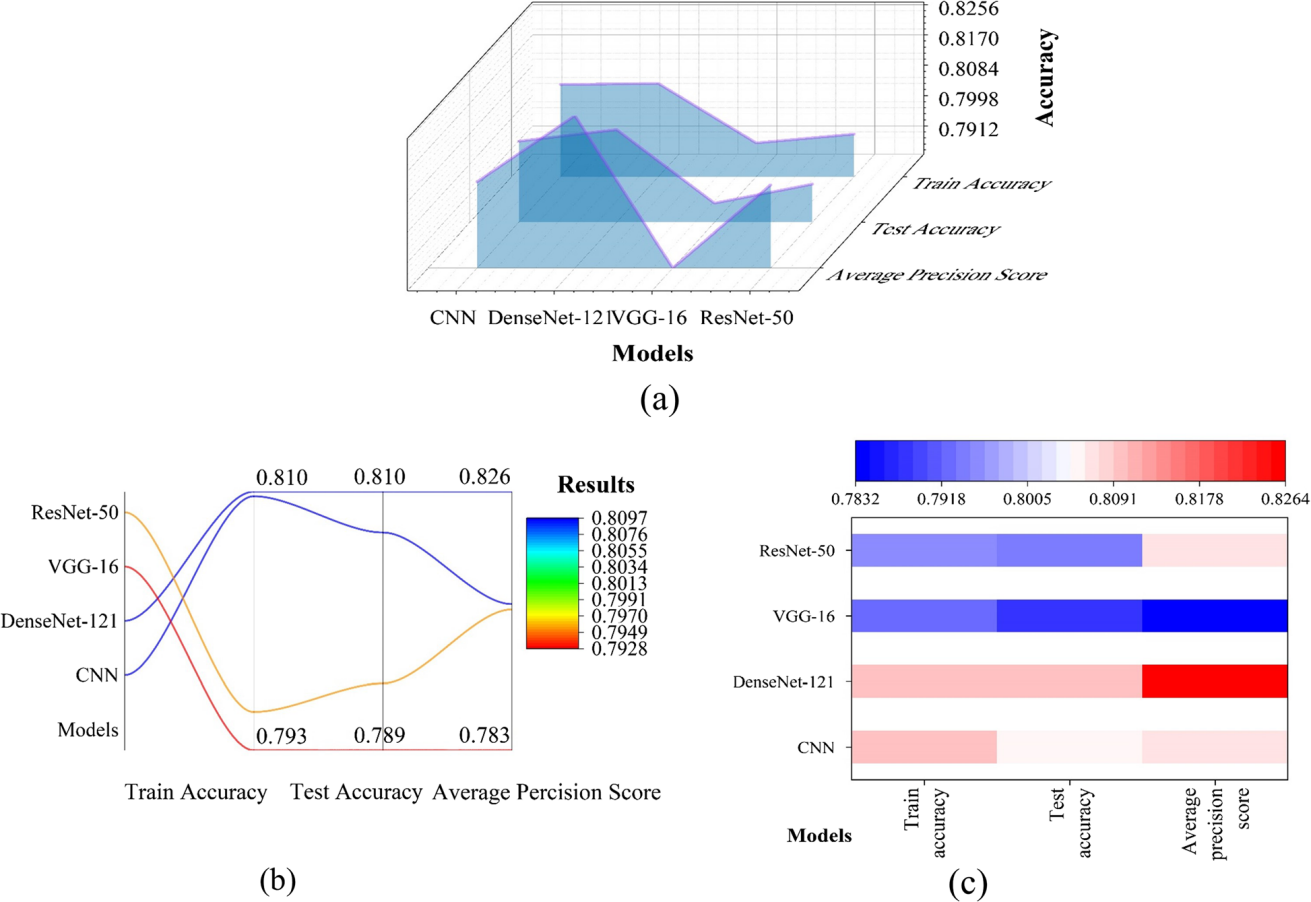
The test accuracy results of CNN, DenseNet, VGG and ResNet

Figure 7 depicts CNN and three transfer learning models (DenseNet-121, VGG-16, and ResNet-50) without labelling. These results represent the mean of ten iterations of the model on the laryngoscopy imaging dataset. According to the results, DenseNet-121 outperformed ResNet-50 and VGG-16. DenseNet outperforms VGG on the ImageNet dataset; this study supports this finding using a difficult laryngoscopy image dataset.

### Accuracy of optimized hybrid structures using proposed indexes and classical indexes

The optimized hybrid structure is combined with convolutional layers, spatial extraction, and vision transformer structures. Four indicators were applied collectively and separately on this optimized hybrid structure. Label-1,2,3,4 indicates that all four indicators were simultaneously applied to the model, whereas Label-1 indicates that only the first indicator was used to identify difficult laryngoscopy, as do Label-2, Label-3 and Label-4. The efficacy of these indicators in identifying difficult laryngoscopy was evaluated using the X-ray dataset, and a ranking order was established.

**Fig. 8:**
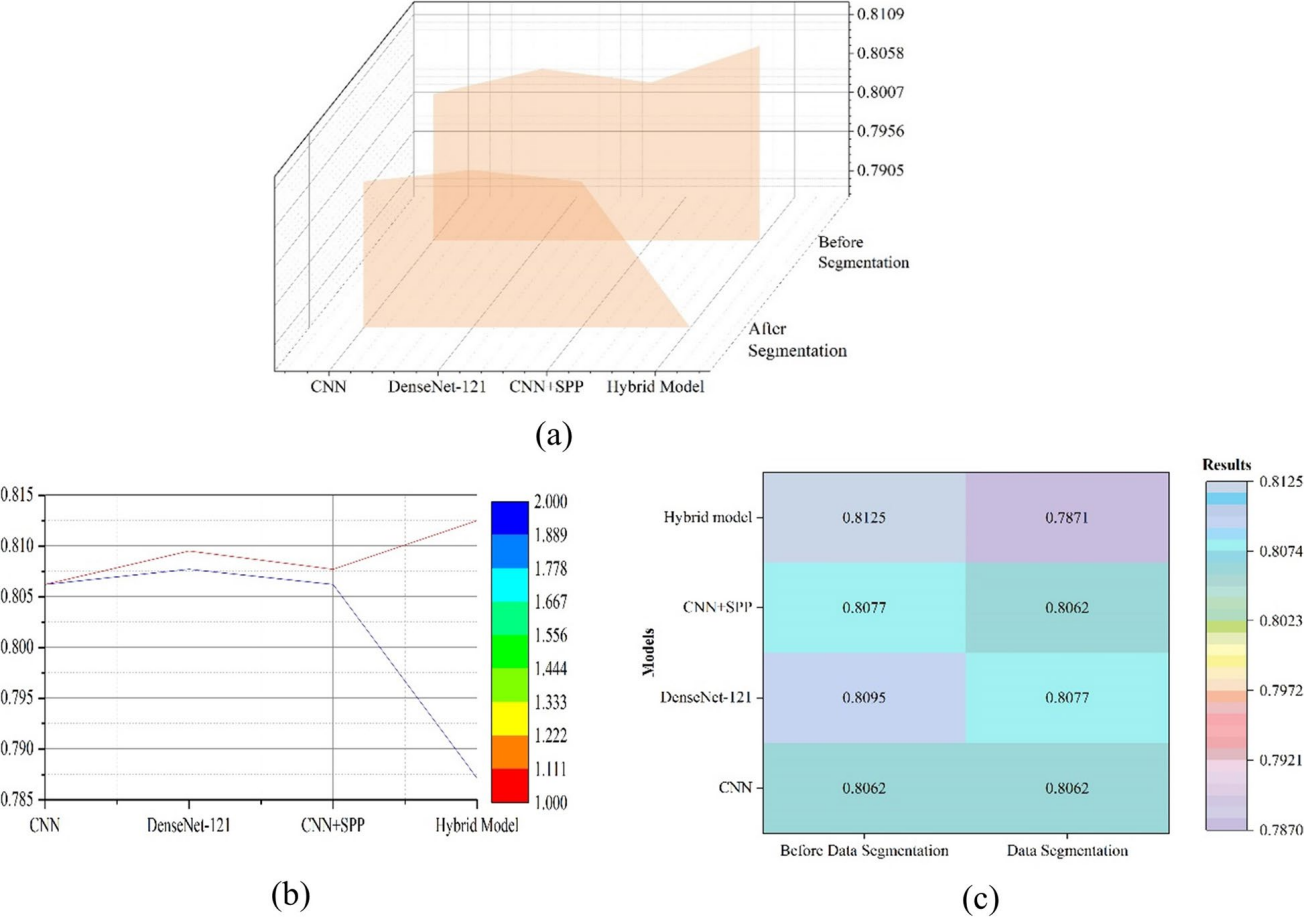
The outcomes of combining CNN, DenseNet-121, and the hybrid model applying segmentation

**Fig. 9:**
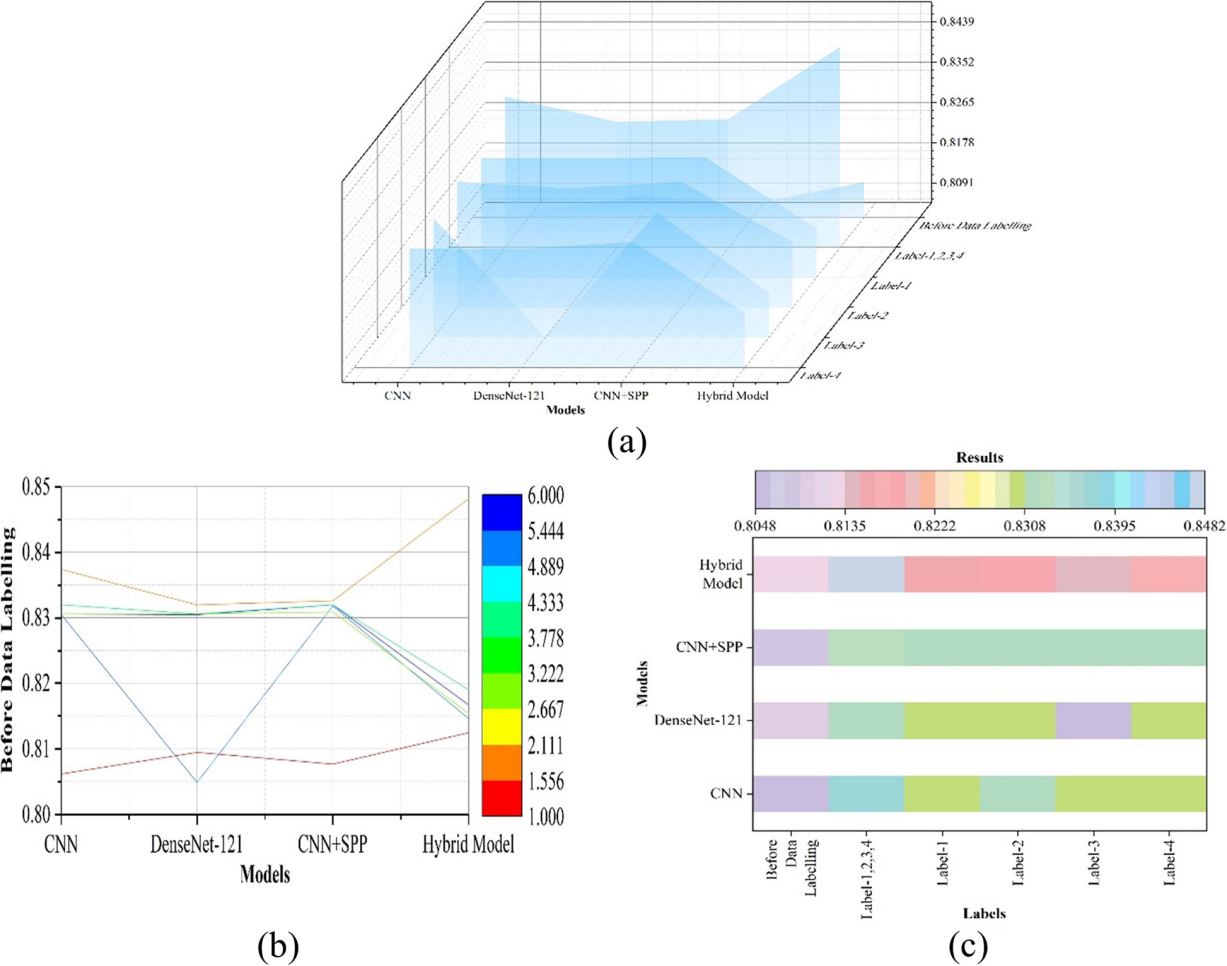
The outcomes of CNN and visual transfer applications using four indicators collectively and individually

**Fig. 10:**
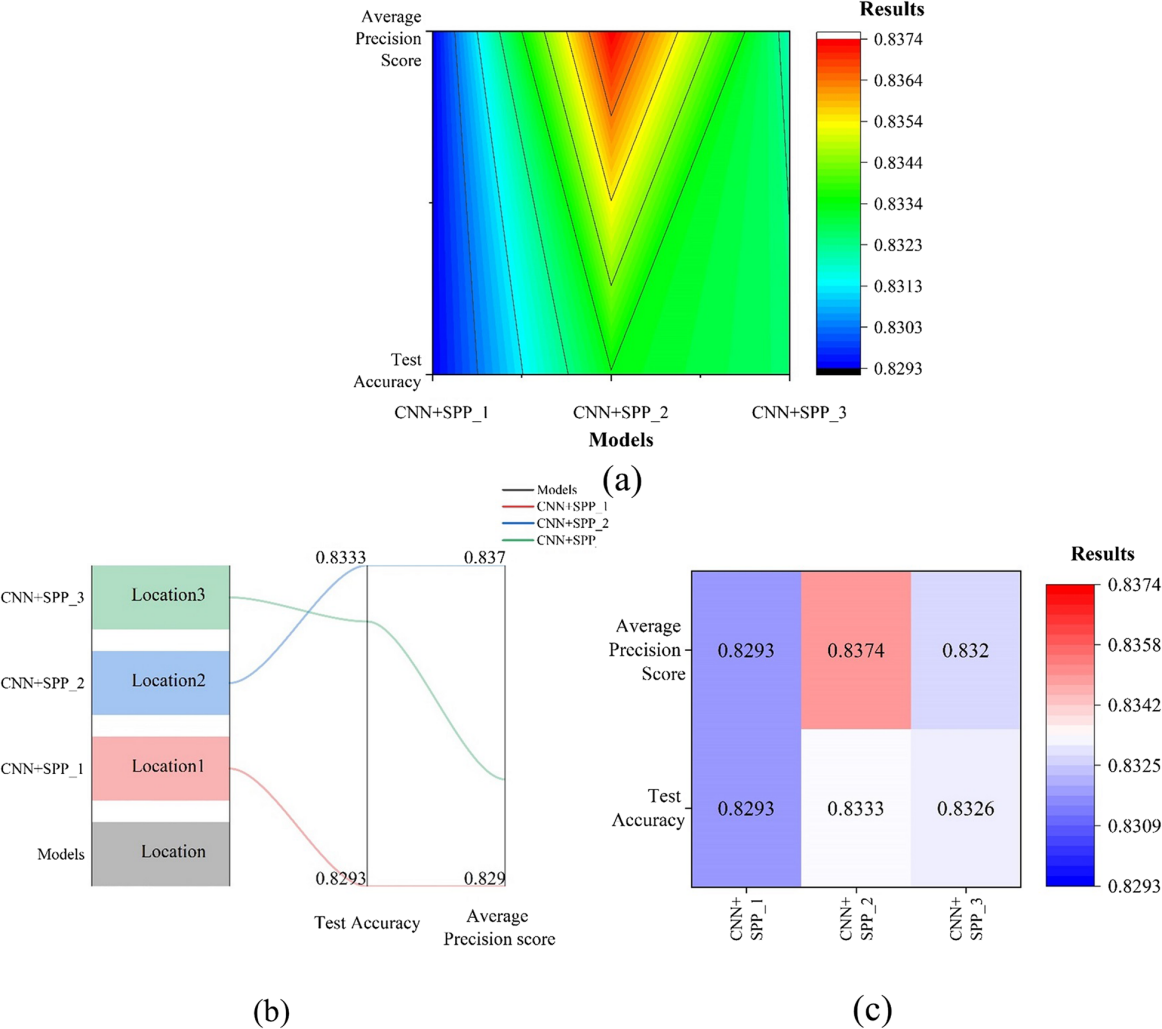
The results of extracting spatial information from different locations. SPP is spatial pyramid pooling

Figure 7 shows the results of CNN, DenseNet-121, and vision transformer applying segmentation. No indicators were labelled on the imaging dataset for difficult laryngoscopy, when applying segmentation. Vision Transformer represents the optimized hybrid structure combined with vision transformer structure in Figs. 7 and 8. The hybrid model achieved the best accuracy of 0.8125 before segmentation. Figure 9 depicts the results of comparing four indicators collectively and separately, as well as simple and hybrid model combined with Vision Transformer. The prediction accuracy of the four indicators were compared separately (0.8309 vs. 0.8320 vs 0.8318 vs 0.8320). The rank of the four indicators was proposed, and Label-2 was the best. Under the combined effect of the four indicators, MSCNN provided the highest level of efficacy, which was 0.8482. Table 2 showed the results of extracting spatial information from different locations. The best place to extract spatial information is to place the spatial extraction structure after each convolutional layer and before batch normalization (Fig. 10). The results showed that the addition of the four indicators improved the performance of difficult laryngoscopy identification.

**Table 2:** The results of extracting spatial information from different locations. SPP is spatial pyramid pooling

Models	Location	Test accuracy	Average precision score
CNN+SPP_1	Location 1: After three convolutional layers	82.93%	82.93%
CNN+SPP_2	Location 2: After each convolutional layer, before Batch Normalization	83.33%	83.74%
CNN+SPP_3	Location 3: After each convolutional layer, after Batch Normalization	83.26%	83.20%

**Table 3:** Clinical predictors of the easy and difficult laryngoscopy groups and their values for predicting difficult laryngoscopy

Items	Easy laryngoscopy group ($n=548$)	Difficult laryngoscopy group ($n=123$)	*P* values	AUC
IIG (cm)	4.4 ± 0.6	4.1 ± 0.6	$<0.001$	0.659
TMD (cm)	L7.9 ± 1.5	7.6 ± 1.7	0.028	0.560
MMT [class I II/class III IV]	371(67.7%)/177(32.3%)	64(52.0%)/59(48.0%)	0.003	0.596

$^a$*Abbreviations:* *IIG* inter-incisor gap, *TMD* thyromental distance, *MMT* modified Mallampati test, AUC area under the curve

Data are presented as mean ± standard deviation or number (proportion, %).

Presently, the popular bedside physical predictors contain inter-incisor gap, thyromental distance and modified Mallampati test. Table 3 displays the performance of the modified Mallampati test in predicting difficult laryngoscopy.

## Discussion

### Proposed indexes

The sufficient prediction of difficult laryngoscopy is considered amongst pivotal priorities for anaesthesiologists in day-to-day work. Nevertheless, the underlying reasons for difficult airways are complicated and there is an absence of gold standards concerning difficult laryngoscopy. Not much research has been done on applying perioperative X-ray databases to differentiate participants with difficult laryngoscopy. This study introduced two new indicators for identification of difficult laryngoscopy: Label-3, the angle between the lower margin of the second cervical spine and the vertical direction, denoting the extent of movement at the higher cervical vertebrae, and Label-4, the angle between the lower margin of the sixth cervical spine and the vertical direction, denoting the extent of movement of the lower cervical vertebrae.

Label-1, the vertical distance from the greatest point of the hyoid bone to the mandibular body, reflects the location of the epiglottis, which has been described by Naguib [37] and Chou [38] among earlier researches. Naguib noticed the absence of a distinction in Label-1 in the difficult laryngoscopy and easy laryngoscopy categories. Nevertheless, Chou discovered Label-1 was lengthier for the difficult laryngoscopy category compared to the easier laryngoscopy category. The test accuracy for Label-1 was 0.8309, suggesting a favourable forecast success. Consistent with the study of Chou HC, Label-1 plays an essential role and produces significant results in predicting difficult laryngoscopy. Horton et al. [39] have proved that the space between the mandible and hyoid is always about 50% of the space between the mandible and the glottis. The large distance between the body of the mandible and the peak of the hyoid bone suggests that the vocal fenestra is profound. In such situations, it was extremely challenging for the anaesthesiologist to reveal the vocal folds due to the presence of tissues in front of the vocal cords. Label-2 is the space separating the occipital bone and the first cervical vertebrae in neutral-positioned intubation participants. Individuals with atlantooccipital damage are at greater risk than normal patients for experiencing difficulties during laryngoscopy [40]. Label-2 is related to the atlantooccipital complex as well as to mandibular prominence. Higher prevalence of distressed airways in those suffering from atlantooccipital composite lesions [40]. The Label-2 has a testing accuracy of 0.8320, indicating a premium level of performance. In addition, the smaller Label-2 length appears to mirror, to some extent, a reduction in movement scope and a slight atlantooccipital joint union. The atlantooccipital was markedly significant in detecting difficult laryngoscopy among the Macintosh laryngoscopy and assistant technical groups during the research.

### Proposed hybrid structure

The proposed MSCNN yielded substantial results. Using MSCNN on a large dataset, the first unified verification of the performance of difficult laryngoscopy identification with a single metric and a combined multimeric was performed. In the study, the predictive accuracies of the four indicators Label-1, Label-2, Label-3, and Label-4 were meaningful (0.8309 vs. 0.8320 vs 0.8318 vs 0.8320). When the four indexes were applied simultaneously, the testing accuracy was 0.8482. The performance of the four indexes, both individually and collectively, was statistically significant. The results showed that the hybrid model reached remarkable levels of performance, surpassing other statistical methods [41] and has the ability to predict difficult laryngoscopy. The usage of four indicators improved the performance of difficult laryngoscopy identification. Jointly and separately, the outcomes of four indicators are comparable, suggesting that clinicians have the clinical discretion to select the most suitable measures for predicting problematic laryngoscopy in clinical practise.The findings demonstrate that it is reasonable to consider substituting alternative indexes when some labelling information in an image dataset is obscured or difficult to annotate due to pose or other factors.

A ranking of the significance of four determinants was presented to assist the anaesthesiologist in recognizing difficult laryngoscopy. As a result, label-2, the atlantooccipital, was discovered to be the most reliable indicator in determining the difficult laryngoscopy outcomes of the research; it behaved marginally better than label-4, the inferior border of the sixth cervical vertebrae in relation to the perpendicular angle, denoting the extent of movement of the inferior cervical vertebrae; followed by label-1, perpendicular to the mandible from the peak of the hyoid bone, then label-3, the corner of the inferior border of the second cervical vertebrae in relation to the perpendicular, showing the extent of movement of the superior cervical spine.

The Mallampati test’s categorization outcome is 0.5960, according to Table 3, this study’s 0.8482 outcome utilizing the MSCNN model is far better than the Mallampati test. The comparison between the simple models and the hybrid model demonstrated that the proposed model enhanced the performance of difficult laryngoscopy identification, and DenseNet-121 outperformed other transferred structures, demonstrating its adaptability to the laryngoscopy database. ImageNet is the pre-trained dataset for all of these transfer learning structures, and it differs significantly from the difficult laryngoscopy databases. For the spatial extraction structure, it is determined that the optimal location for extracting spatial information is to position the spatial-extraction structure after each convolution phase and before batch normalisation. Future emphasis will be placed on pre-training transfer learning structures on open-source medical datasets such as Medmnist [42]. Further work will also concentrate on the comparison of a wider variety of metrics related to the difficult laryngoscopy categorization, both separately and holistically.

## Conclusion

Causes of difficult laryngoscopy vary widely and there is no gold standard. The first unified validation of difficult laryngoscopy decision-making under both singular and combined multi-indicators is presented. This study demonstrated the reliability and efficacy of two new indicators related to upper and lower cervical motion for identifying difficult laryngoscopy. The efficacy of predicting difficult laryngoscopy was improved by combining two new and two established indicators.The classification of the four indicators independently revealed that the atlantooccipital clearance was slightly better than the other indicators. The MSCNN method outperforms all the other methods in difficult laryngoscopy prediction most of the time. The proposed MSCNN supported by deep learning, spatial extraction and vision transformer structures, enables effective and dependable predictions of difficult laryngoscopy.

## Data Availability

The datasets used and analysed during the current study are available from the corresponding author on reasonable request. The data that support the findings of this study are available from Peking University Third Hospital but restrictions apply to the availability of these data, which were used under license for the current study, and so are not publicly available. Data are however available from the authors upon reasonable request and with permission of Peking University Third Hospital.
